# Early and late complications following hematopoietic stem cell transplantation in pediatric patients – A retrospective analysis over 11 years

**DOI:** 10.1371/journal.pone.0204914

**Published:** 2018-10-16

**Authors:** Sophie Hierlmeier, Matthias Eyrich, Matthias Wölfl, Paul-Gerhardt Schlegel, Verena Wiegering

**Affiliations:** University Hospital Wuerzburg, Children’s Department of Oncology, Hematology and Stem Cell Transplantation, Wuerzburg, Germany; Children's Hospital Boston, UNITED STATES

## Abstract

Hematopoietic stem cell transplantation (HSCT) has been an effective method for treating a wide range of malignant or non-malignant disorders. In case of an autologous HSCT, patients receive their own stem cells after myeloablation before extraction. Allogeneic HSCT uses stem cells derived from a donor. Despite being associated with a high risk of early and long-term complications, it is often the last curative option. 229 pediatric patients, who between 1 January 2005 and 31 December 2015 received an HSCT at the University Children’s Hospital Wuerzburg, were studied. Correlations between two groups were calculated with the Chi square test or with a 2x2-contingency table. To calculate metric variables, the Mann-Whitney-U-test was used. Survival curves were calculated according to Kaplan and Meier. Significance was assumed for results with a p-value <0.05 (CI (Confident Interval) 95%). We retrospectively analyzed 229 pediatric patients (105 females, 124 males) for early and late complications of allogeneic and autologous hematopoietic stem cell transplantation. Median age at HSCT was seven years. Underlying diseases were leukemia (n = 73), lymphoma (n = 22), solid tumor (n = 65), CNS (central nervous system)- tumor (n = 41), and “other diseases” (n = 28). Survival times, overall survival, and event-free survival were calculated. Of all patients, 80.8% experienced complications of some degree, including mild and transient complications. Allo-HSCT (allogeneic HSCT) carried a significantly higher risk of complications than auto-HSCT (autologous HSCT) (n = 118 vs. n = 67; p = < .001) and the remission rate after allo-HSCT was also higher (58.7% vs. 44,7%; p = .032). Especially infection rates and pulmonary complications are different between auto- and allo-HSCT. Leukemia patients had the highest risk of early and late complications (95,0%; p < .001). Complications within HSCT are major risk factors following morbidity and mortality. In order to detect complications and risk factors early, strict recordings are needed to reduce the rate of complication by recognition and prevention of triggering factors. In the future, these factors should receive greater attention in the planning of HSCT post-transplantation care in order to improve the results of the transplantation and establish protocols to prevent their occurrence.

## Introduction / Background

Hematopoietic stem cell transplantation (HSCT) is an effective treatment for certain childhood cancers, diseases of the hematopoietic system, or autoimmune diseases.[1] First performed successfully in the 1970s, it is now an established therapy.[2] Worldwide, 50.000 HSCT are performed annually with survival rates exceeding 80%.[3, 4] Main indications for HSCT are leukemia, refractive lymphoma, solid tumor, central nervous system (CNS) tumor, and non-malignant diseases like metabolic, autoimmune–for example T1D[5]–or hematopoietic disorders.[6, 7] The prior chemo- (and radiation) therapy plus transplantation itself can cause various complications that contribute to the relatively high morbidity and mortality rates.[8] Transplantation-associated morbidity and mortality rates have declined significantly in recent years due to advances in transplantation medicine with tailored conditioning regimens, precise HLA (human-leucocyte-antigen) -typing, improved supportive therapy, and prophylaxis against severe infections.[9] Further reduction of the complication rate to improve outcomes following HSCT requires detailed therapy and follow-up care protocols tailored to each patient’s risk factors. Our relatively heterogeneous patient collective reflects real pediatric oncological clinical practice in use of stem cell transplantation. The present retrospective study should help to identify prognostic markers, provide guidance for follow-up measures in the future, and support individualized stem cell transplantation strategies in order to ameliorate short and long-term-toxicities.

## Subjects and methods

### Patients and data management

A total of 229 pediatric patients, who underwent HSCT between 1 January 2005 and 31 December 2015 at the University Children Hospital Wuerzburg, were studied. Patient data was obtained from the patient registry SAP and from patient files and was then recorded in Microsoft Excel files (S1 Table) (Microsoft Office Excel 2011). Endpoint was 31 December 2015 or the date of a patient’s death. The study was conducted solely based on archived data. All patients approved the retrospective analysis of their data. The declaration of clearance of the Ethics Committee of the Faculty of Medicine, Julius-Maximilians-University Wuerzburg has been obtained. The Ethics Committee concluded that there are no ethical and legal aspects of the statistical evaluation of our data (S1 Fig).

### Study objectives

In our retrospective, we analyzed complications of the therapeutic process and long-term-effects after HSCT in pediatric patients. We analyzed individual patients after autologous and allogeneic HSCT and these two groups of patients comparatively to find differences. In order to detect complications and risk factors early, strict recordings are needed to reduce the rate of complication by recognition and prevention of triggering factors. In the future, these factors should receive greater attention in the planning of HSCT post-transplantation care in order to improve the results of the transplantation and establish protocols to prevent their occurrence.

### Statistical analysis

Statistical evaluation was done with IBM SPSS Statistics 23 Premium 02 for Mac. Correlations and differences between two groups were calculated with the Chi-square test. The Chi-square test is a nonparametric test that is used to compare more than two variables for a randomly selected data. Low cell frequency variables were calculated with a 2x2-contingency table using Fisher’s exact test. To calculate the significance of metric variables, the Mann-Whitney-U-test was used for non-parametric values. Survival curves were calculated according to Kaplan and Meier. These analytic methods enabled calculation of survival times, overall survival, and event-free survival. Significance was assumed for results with a p-value <0.05 (CI 95%).

### Patient cohort

For our statistical analysis following groups were formed:

Group 1: Entire cohort: All patients who have undergone stem cell transplantation during the observation period.
Group 2: Separate analysis allogeneic vs. autologous HSCT.
Group 3: Statistical evaluation of allogeneic patients.

## Results

### Patient and stem cell characteristics

During the 11-year study period, 229 pediatric patients (105 females, 124 males; median age was 7 y [SD (Standard deviation) ± 6.56, IQR (Interquartile range) 13]) underwent HSCT. The underlying disease in 31.7% (n = 73) of patients was leukemia, in 9.6% (n = 22) lymphoma, in 28.3% (n = 65) solid tumor, in 17.8% (n = 41) CNS tumor, and in 12.2% (n = 28) “other diseases” that are non-malignant-diseases such as WAS (Wiskott-Aldrich-Syndrome), HLH (Hemophagocytic Histiocytosis), Blackfan-Diamond-Syndrome, Omenn-Syndrome, LCH (Langerhans cell histiocytosis), Congenital dyserythropoietic anemia, SCID (Severe combined immunodeficiency), Kostmann disease, thalassemia, X-ALD (X-linked-Adrenoleucodystrophy), Glanzmann’s thrombastenia, and aplastic anemia. 85.6% (n = 196) of patients received prior chemotherapy or irradiation. All patients received myeloablative conditioning. After HSCT, 24.9% (n = 57) of patients were re-transplanted (auto-auto-HSCT 11.4% [n = 26]; auto-allo-HSCT 1,7% [n = 4]; allo-allo-HSCT 5.2% [n = 12]; >2 transplantations in 3.1% [n = 7]). 18.3% (n = 23) of allo-HSCT patients received a DLI (Donor lymphocyte infusion), and 8.7% (n = 20) of all patients had follow-up irradiation. The median follow-up for all patients was 834.4 days (SD ±947.63 d (days)) (Table 1).

**Table 1:** Patient & stem cell charasteristics.

Patient & stem cell characteristics	Total number of patients (n(%))	Allogeneic HSCT: total: n (%)	Autologous HSCT: total: n(%)	p-value
**Number of patients**	**229 (100)**	**126 (100)**	**102 (100)**	
**Gender distribution**				**.047**^FE^*
Female	105 (45.9)	51 (40.5)	54 (52.4)	
Male	124 (54.1)	75 (59.5)	49 (47.6)	
**Underlaying disease**				**< .001** ^**Chi2**^ *
Leukemia	73 (31.9)	73 (57.9)	0 (0.0)	
Lymphoma	22 (9.6)	12 (9.5)	10 (9.7)	
Solid tumor	65 (28.3)	13 (10.3)	52 (50.5)	
CNS tumor	41 (17.8)	0 (0.0)	41 (39.8)	
Other disease	28 (12.2)	28 (22.2)	0 (0.0)	
**Previous therapy**				
Pre-therapy	196 (85.6)	93 (73.8)	103 (100.0)	< .**001** ^**Chi2**^ *
No-pre-therapy	33 (14.4)	33 (26.2)	0 (0.0)	**< .001**^Chi2^*
Only chemotherapy	114 (49.8)	41 (32.5)	73 (70.9)	**< .001**^Chi2^*
Chemotherapy combined with irradiation	77 (33.6)	48 (38.1)	29 (28.2)	.124^Chi2^
**Age at HSCT**				**.045** ^**MWU**^ *
Average	8.50	9.39	7.42	
Median	7.00	9.00	5.00	
Standard deviation	6.560	6.826	6.077	
IQR	13	12	11	
**Post-HSCT treatment**				
Re-transplant	57 (24.9)	13 (10.3)	44 (42.7)	**< .001** ^**EF**^ *
Donor lymphocyte infusion	23 (10.0)	23 (18.3)	0 (0.0)	—
Irradiation	20 (8.7)	7 (5.6)	13 (12.6)	.098 ^EF^
**Outcome**				**.032** ^**FE**^ *
Remission	123 (53.7)	74 (58.7)	49 (47.6)	
Transplant-related mortality				
whithin 100d after HSCT	9 (3.9)	2 (1.6)	7 (6.8)	
whithin 1year after HSCT	24 (10.5)	11 (8.7)	13 (12.6)	
whithin 2years after HSCT	25 (10.9)	13 (10.3)	12 (11.7)	
Alive with disease	29 (12.6)	11 (8.7)	18 (17.5)	
Died of disease	47 (20.5)	23 (18.3)	24 (23.3)	
Other cause of death	5 (2.9)	5 (4.0)	0 (0.0)	
**Stem cell source**				**.<001** ^**FE**^ *
Peripheral blood	205 (89.5)	102 (81.0)	103 (100.0)	
Bone marrow	22 (9.6)	22 (17.5)	0 (0.0)	
Cord blood	2 (0.9)	2 (1.5)	0 (0.0)	

Table 1 shows patient and stem cell characteristics in comparison of course and outcome in allogeneic versus autologous HSCT patients with p-values and statistical tests used. Chi^2^ = Chi-square-Test; FE = Fisher’s exact test; MWU = Mann-Whitney-U-test. CI 95%; P≤0.

* Significant at the 0.05 probability level.

### Source and donor

**Fig 1:**
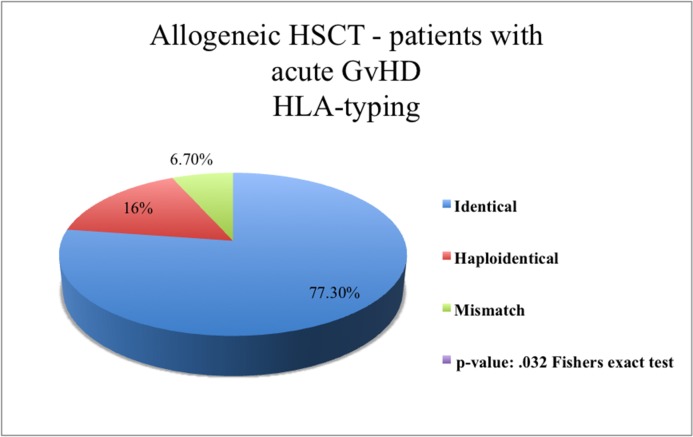
Patients after allogeneic HSCT with acute GvHD. Stated the HLA-typing of transferred stem cells. The distribution of HLA-typing across all patients in the allogeneic patient group occurred acute GvHD is displayed as a pie chart. Statistical significant difference p = .002^FE^.

**Fig 2:**
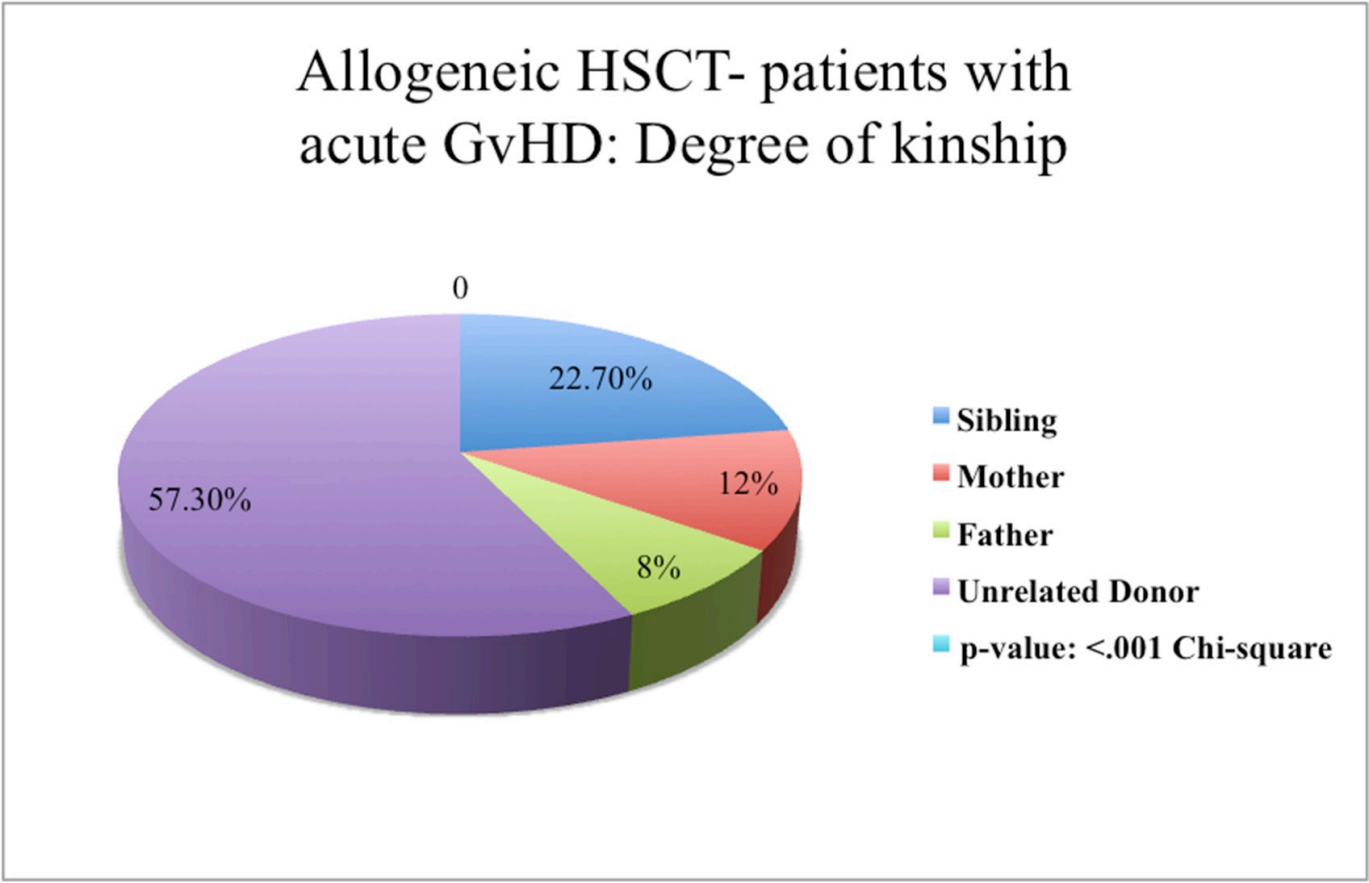
Patients after allogeneic HSCT with acute GvHD. Stated the degree of kinship between donor and receiver of stem cells. The distribution of degree of kinship across all patients in the allogeneic patient group occurred acute GvHD is displayed as a pie chart. Statistical significant difference p < .001^Chi2^.

As a source of stem cell, grafts mobilized peripheral blood cells, bone marrow cells, and cord blood stem cells were used in 89.5% [n = 205], 9.6% [n = 22], and 0.9% [n = 2] of patients respectively (Table 1). 81.0% [n = 102] of our allo-HSCT patients received HLA-identical stem cells and 11.1% [n = 14] received haploidentical stem cells. In 7.9% [n = 10] of cases, HLA-mismatched HSCT (9/19) were transplanted. 18.3% [n = 23] of patients received stem cells from an HLA-compatible sibling and 70.6% [n = 89] received stem cells from an unrelated donor (UD). In 7.1% [n = 9] of patients, the patient’s mother and in 4.0% [n = 5] of patients, the patient’s father was the stem cell donor (Fig 1, Fig 2).

### Allo-HSCT vs. auto-HSCT

**Fig 3:**
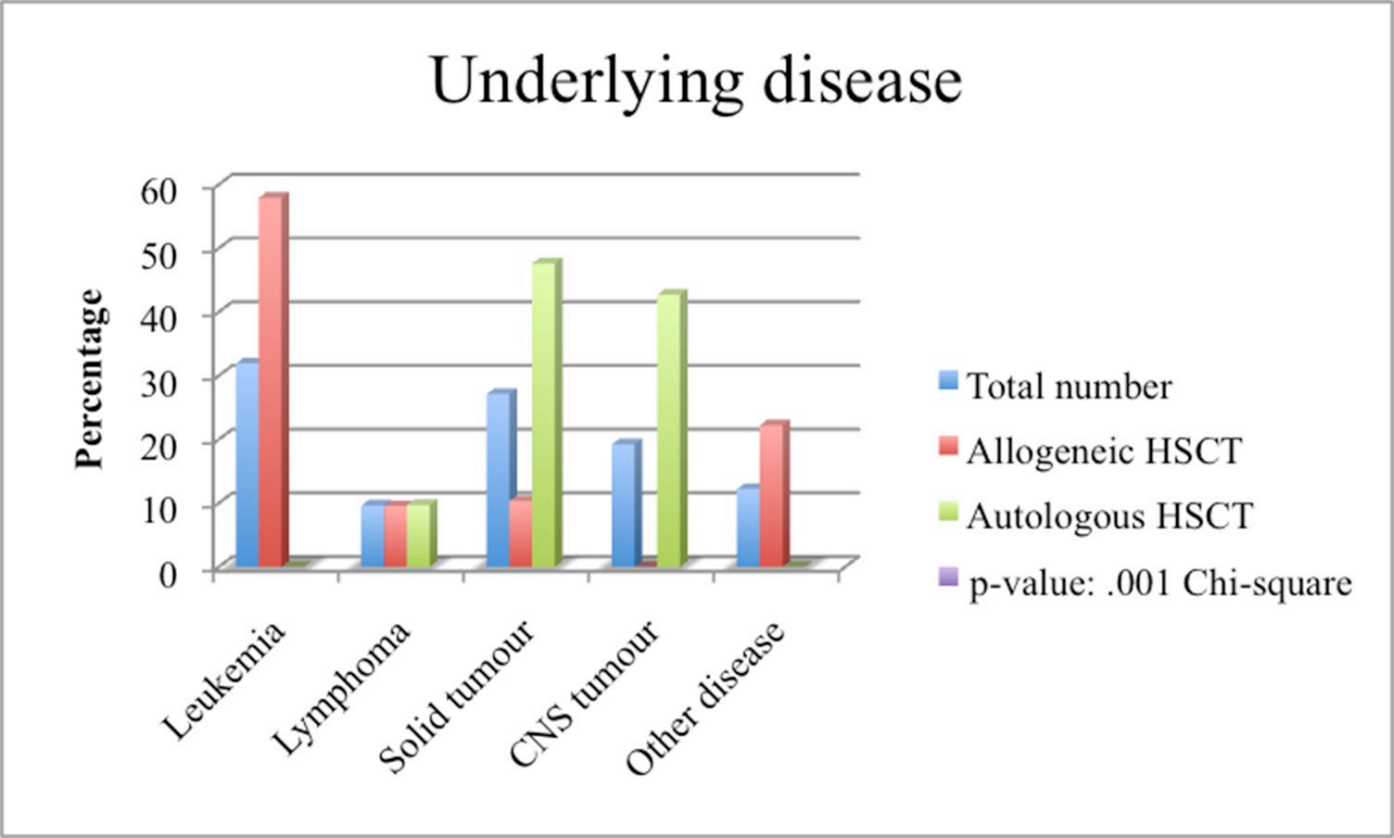
Underlying diseases in the entire cohort and a comparison between allogeneic and autologous HSCT. The distribution of underlying disease across all patients and in allogeneic and autologous patient groups is displayed as a bar chart. Statistical significant difference: p < .001 ^Chi2^.

55.0% (n = 126) of patients received allo-HSCT, 45.0% (n = 103) auto-HSCT. Allo-HSCT patients were more often male (59.5%, n = 75) than female (40.5%, n = 51), as were auto-HSCT patients (52.4% [n = 54] male vs. 47.6% [n = 49] female) (p = .047). As expected, the distribution of the underlying diseases between the two groups was different (p = < .001) (Table 1, Fig 3). 100.0% (n = 103) of auto-HSCT patients and 73.8% (n = 90) of allo-HSCT patients had received prior chemotherapy and/or irradiation (p = < .001) (Table 1)

### Complications

**Fig 4:**
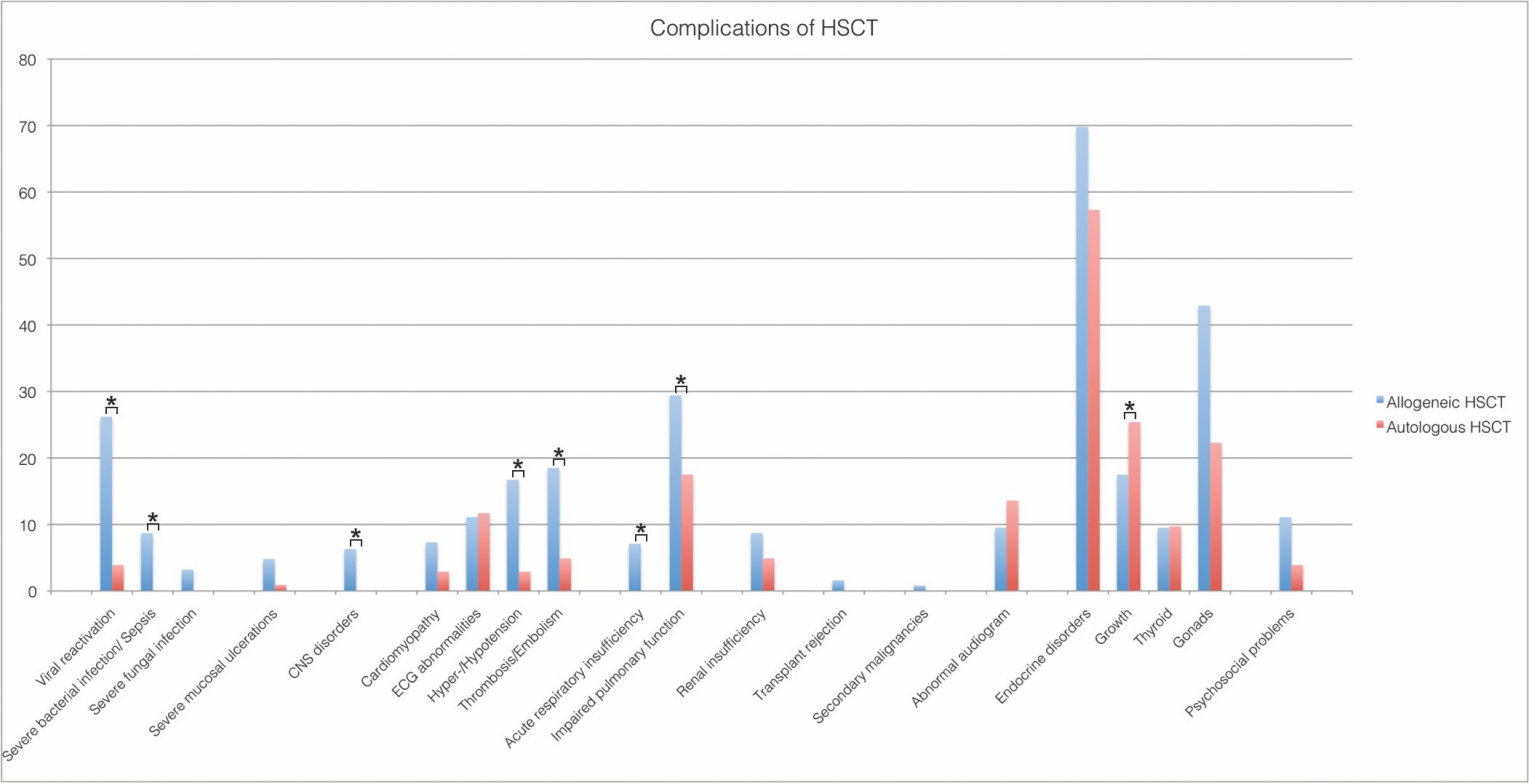
Complications of HSCT. Percentage allocation of specific complications in allogeneic (n = 126; overall complication rate 88.9%) and autologous (n = 103; overall complication rate 56.3%) patients (p < .001). The distribution of specific complications across all patients with complications is displayed as a bar diagram. Statistical significant differences (p ≤ 0.05) for the comparison between allogeneic and autologous HSCT as detected by pair-wise Chi-Square test for viral reactivation, severe bacterial infection, impaired pulmonary function, in endocrine disorders: growth retardation and Fisher’s exact test for acute respiratory insufficiency, thrombosis/embolism, hyper-/hypotension and CNS disorders, are marked with asterisks (*).

Of all patients, 74.2% (n = 175) suffered of early or late complications (S2 Table), 25.8% (n = 59) were free of complications. Complications were assessed according to the type of transplantation and the underlying disease. More complications occurred after allo-HSCT than after auto-HSCT (88.9% [n = 112] vs. 56.3% [n = 58]) (p = < .001). In the allo-setting, we found a greater risk of severe bacterial infection/sepsis (8.7% [n = 11] vs. 1.0% [n = 1] (p = .014) as well as viral reactivations (26.2 [n = 33] vs. 2.9 [n = 3] (p = < .001). Furthermore, pulmonary insufficiency (eight patient required intensive care unit management in the allo-transplant group), and later on impaired respiratory function, were also more common after allo-HSCT. Further details are listed in Table 2/ Fig 4. Patients with leukemia had the greatest risk of complications (91.8% [n = 67]; p < .001). In detail, 11.0% [n = 8] (p.020) suffered from respiratory insufficiency. Hyper-/hypotension, requiring medication, was seen in 17.8% [n = 13] (p.006) and psychosocial problems, requiring medication, was seen in 13.7% [n = 10] (p = .001). Furthermore, the majority of patients with complications of the hypothalamic-pituitary/gonodal axis were lymphoma patients (68.0% [n = 15] (p = .003)). Significant differences in the rates of complication were found in re-transplanted patients: allo-allo-HSCT 63.6% [n = 8] (p = .007), auto-allo-HSCT 100% [n = 4] (p = .021), whereas tandem autologous HSCT did not increase the risk of complications. In relation to medications given as a part of the conditioning, the following statistically significant connections were found: 36.7% [n = 10] (p = .010) of the patients with thrombosis/embolism after HSCT got Bu/Mel-based (Busulfan/Melphalan) conditioning. Patients with previous TBI (Total body irradiation) showed an increased incidence of pulmonary impairment (lung dysfunction): 43.6% [n = 24] (p = .005), CNS-disorders: 62.5% [n = 5] (p = .005), ECG (Electrocardiogram) abnormalities: 26.9% [n = 7] (p = .<001) as well as thrombosis/embolism: 42.9% [n = 12] (p = .<001). 28.6% [= 42] (p = .045) of patients, who had received platin-agent based conditioning, showed endocrine disorders.

**Table 2:** Complications after HSCT.

Complications of HSCT	Total patients: n (%)	Allogeneic HSCT: n (%)	Autologous HSCT: n (%)	p–value
	229 (100)	126 (100)	103 (100)	
**Complications**				
yes	170 (74.2)	112 (88.9)	58 (56.3)	
no	59 (25.8)	14 (11.1)	45 (43.7)	
**Infections**				
Viral reactivation	36 (16.2)	33 (26.2)	3 (2.9)	**< .001** ^**Chi2**^ *
chronic	4 (1.7)	4 (3.2)	0 (0,0)	**< .001** ^**Chi2**^
transient	32 (13.5)	28 (22.3)	4 (3.8)	
Severe bacterial infection/Sepsis	12 (5,2)	11 (8,7)	1 (1,0)	.**014** ^**Chi2**^ *
Severe fungal infection	4 (1,7%)	4 (3,2)	0 (0,0)	.129^Chi^
**Severe mucosal ulcerations**	7 (3.1)	6 (4.8)	1 (1,0)	.133 ^FE^
**CNS disorders**	8 (3,4)	8 (6,3)	0 (0.0)	.**009** ^**FE**^ *
**Cardio-vascular complications**				
Cardiomyopathy	13 (5.7)	10 (7.3)	3 (2.9)	.151 ^Chi2^
ECG abnormalities	25 (10.9)	13 (10.3)	12 (11.6)	.734 ^FE^
Hyper—/ Hypotension	24 (10.5)	21 (16.7)	3 (2.9)	**< .001** ^**FE**^ *
Thrombosis / Embolism	29 (12.7)	24 (19.0)	5 (4.9)	**.001** ^**Chi2**^ *
**Pulmonary complications**				
Acute respiratory insufficiency	9 (3.9)	9 (7.1)	0 (0.0)	**.005** ^**FE**^ *
Impaired pulmonary function	55 (24.0)	37 (29.4)	18 (17.5)	.**043** ^**Chi2**^ *
**Renal insufficiency**	16 (7.0)	11 (8.7)	5 (4.9)	.252 ^Chi2^
**Transplant rejection**	2 (0.9)	2 (1.6)	0 (0.0)	.503 ^FE^
**Secondary malignancies**	1 (0.4%)	1 (0.8)	0 (0.0)	1.000 ^FE^
**Abnormal audiogram**	26 (11.4)	12 (9.5)	14 (13.6)	.611 ^Chi2^
**Endocrine disorders**				
Total number	116 (50.7)	70 (55.6)	46 (44.7)	.112 ^Chi2^
Growth	33 (14.4)	14 (11.1)	19 (18.4)	.132 ^Chi2^ *
Thyroid	22 (9.6)	12 (9.5)	10 (9.7)	1.000 ^FE^
Gonads	85 (38.4)	54 (42.9)	31 (30.1)	.055 ^Chi2^
**Psychosocial problems**	18 (7.8)	14 (11.1)	4 (3.9)	.**043** ^**Chi2**^

Table 2 shows complications during the course of HSCT. Comparison of allogeneic and autologous HSCT patients with p-values and statistical tests used.

*Calculation of the p-value stating used tests: Chi^2^ = Chi-square-Test; FE = Fisher’s exact test. CI 95%; P≤0.05. * Significant at the 0.05 probability level.

### GvHD as complication and risk factor

Among all allo-HSCT patients (n = 126), 59.5% (n = 75) developed an aGvHD (acute Graft-versus-Host-Disease) (any grade). Skin was the organ chiefly affected (92.0% [n = 69]), followed by the GI-tract (Gastro-intestinal-tract) (20.0% [n = 15]), and the liver (14.7% [n = 11]). 84.3% of these patients (n = 64) had severity grades I or II, 12.0% (n = 9) grade III, 4.0% (n = 3) grade IV. 60.0% (n = 45) of patients with leukemia had aGvHD (p < .001); 21.3% (n = 16) of them had received non-HLA-identical cell (haploidentical/mismatch) transplants (p = .002), while 66.7% (n = 50) had received transplants from an unrelated donor (UD) (p < .001). 74.7% (n = 56) of transplants were not manipulated prior to the transplantation (p = .013) (detailed in Table 3).

**Table 3:** Allogeneic patients with GvHD after HSCT.

GvHD in allogeneic HSCT patients: total number: n = 126 (100%)			p–value*
	Acute GvHD: n = 75 (59.5%)	Chronic GvHD: n = 28 (22.2%)	
**Organ involvement**			
Skin	69/75 (92.0)	23/28 (39.3)	
Intestine	15/75 (20,0)	6/28 (21.4)	
Liver	11/75 (14.7)	2/28 (7.1)	
Others	1 /75(1.3)	3 /28 (13.0)	
**Classification**	**Maximum severity**	**Classification**	
	I: 40/75 (53.3) II: 23/75 (30.7)	local 16/28 (57.1) limited 9/28 (32.1)	
	III: 9/75 (12.0)	extended 3/28 (10.7)	
	IV: 3/75 (4.0)		
Underlying disease			**aGvHD: <001**^**Chi2**^ * cGvHD: < .001^**EF**^ *
Leukemia	44 (58.7)	15 (65.2)	
Lymphoma	4 (5.3)	2 (8.7)	
Solid tumor	9 (12.0)	1 (4.3)	
CNS tumor	0 (0.0)	0 (0.0)	
Other disease	18 (24.0)	5 (21.7)	
**Acute GvHD**	**Number of patients: 75**		
**HLA-typing**			**.002** ^**FE**^ *
Identical	59 (78.7)		
Haploidentical	10 (13.3)		
Mismatch 9/10	6 (8.0)		
**Degree of kinship**			**< .001**^**FE**^ *
Sibling	14 (18.7)		
Mother	6 (8.0)		
Father	5 (10.7)		
UD	50 (66.7)		
**Graft manipulation**			**.013** ^**FE**^ *
No graft-manipulation	56 (74.7))		
CD-34^+^-selection	4 (5.3)		
Depletion of CD3^+^/CD19^+^-cells	12 (16.0)		
Depletion of TCRαβ/CD19^+^-cells	1 (1.3)		
Partial T-cell depletion	2 (2.7)		
**Chronic GvHD**	**Number of patients: 28**		
**HLA typing**			.168^FE^
Identical	24 (85.7)		
Haploidentical	1 (3.6)		
Mismatch (9/10)	3 (10.7)		
**Degree of kinship**			**< .001**^**FE**^
Sibling	5 (21.7)		
Mother	1 (4.3)		
Father	0 (0.0)		
UD	17 (73.9)		
**Graft manipulation**			195^FE^
No graft-manipulation	25 (89.3)		
CD-34^+^-selection	0 (0.0)		
Depletion of CD3^+^/CD19^+^-cells	3 (10.7)		
Depletion of TCRαβ/CD19^+^-cells	0 (0.0)		
Partial T-cell depletion	0 (0.0)		

Table 3 presents allogeneic HSCT patients with acute or chronic GvHD showing affected organ, classification of GvHD, the underlying disease, and stem cell characteristics with p-values and statistical tests used. Calculation of the p-value stating used tests: Chi^2^ = Chi-square-Test; FE = Fisher´s exact test. CI 95%; P≤0.05.

22.2% (n = 28) of patients developed a cGvHD (chronic Graft-versus-Host-Disease) after allo-HSCT. Again, skin was the most commonly affected organ with 82.1% (n = 23), followed by the GI-tract (28.6% [n = 8]), and liver the (10.7% [n = 3]; (p < .001)) Local cGvHD (mild) showed in 57.1% [n = 16], limited cGvHD (moderate) in 32.1% [n = 9]. 10.7% [n = 3] of our patients had extended cGvHD (severe) (Table 3).

Like aGvHD, cGvHD most often affected leukemia patients (67.9% [n = 19]; (p = < .001)). 14.3% (n = 4) of patients received non-HLA-identical transplants (n.s.), 75.0% (n = 21) received cells from a UD (n.s.). In 82.6% (n = 25) of cases, the transplant had not been manipulated (n.s.) (Table 3).

## Discussion

Early and late complications after HSCT are associated with high morbidity and mortality rates in children. In the present study we describe these complications in a cohort of children aged seven years (median) at HSCT. Although data on complications after HSCT in children are sparse, they suggest that they somewhat differ from complications in adults. Therefore, we retrospectively analyzed this patient cohort in order to facilitate development of preventative approaches for patient conditioning and protocols for follow-up care that minimize the risk of complications.

Many of the complications after HSCT involve viral, bacterial, and fungal infections. Explicit guidelines exist to lower the risk of these infections.[10, 11] Severe bacterial or fungal infections/sepsis were detected in 8.7% of our allo-HSCT patients and only in 1.0% of patients after auto-HSCT. These pathogens chiefly affected patients after allo-HSCT due to the longer time it took to complete immune reconstitution of mainly neutrophilic granulocytes (16 d vs. 11 d). The higher numbers can be explained by the longer time it took to rebuild a well-functioning immune status after allo-HSCT.

Severe post-transplant mucous membrane ulceration was documented in 4.8% of our allo- and in 1.0% of our auto-HSCT patients. For this analysis early mucositis based on the conditioning regimen, which sometimes can be significant, was excluded. In our patients, mucositis after HSCT results from infectious pathogens (mostly the Herpes virus). In addition to antibiotics, possible treatments include growth factors such as G-CSF (Granulocyte-colony-stimulating factor), GM-CSF (Granulocyte-Monocyte-CSF), or interleukins.[12]

CNS disorders–in our cohort clinical symptoms of PRES (Posterior reversible encephalopathy syndrome) or radiologic proved pathologies–were observed in 6.3% of allo-HSCT patients post-HSCT. The incidence of CNS disorders reported in the literature varies widely from 1.6% to 15.4%. A common cause is PRES,[13] which was documented as the cause in 75% of our patients with CNS disorders. As previously reported by Bresters and colleagues, CNS complications were experienced by 35.6% of their patients. In comparison to our study, the higher incidence can be explained by a younger age at HSCT (under 3y) and a longer period of follow-up (10.4y), compared to median age at HSCT 7y and median follow-up of 2.3y.

Acute respiratory insufficiency/dyspnoea affected 7.1% of our allo-HSCT patients, 88.9% (8 patients) of them requiring mechanical ventilation in an Intensive care unit. The triggering factor for respiratory insufficiency or failure is Acute respiratory distress syndrome, bacterial pneumonia, or sepsis. Studies show a high mortality rate in HSCT patients needing intubation.[14, 15] Of our patients, 62.5% (n = 5) of those requiring mechanical ventilation died. A study that investigated the mortality of pediatric HSCT patients found that 18.4% required intensive care, of which 88.5% of needed mechanical ventilation. The mortality rate in that study was 57.7%, similar to our rate of 62.5%.[16] One late complication in HSCT patients is abnormal spirometry secondary to pulmonary dysfunction: 24.0% of our patients had pathologic pulmonary function, significantly more patients after allo-HSCT (29.4%) than after auto-HSCT (17.5%). The risk factors here are infections, pulmonary complaints prior to HSCT, aGvHD, and TBI.[17, 18] Our patients with aGvHD or prior TBI were at an increased risk. The discrepancy between allo-HSCT and auto-HSCT patients can be explained by the association of pulmonary abnormalities with GvHD seen only in allo-HSCT patients.

Many drugs given to patients who subsequently undergo HSCT have nephrotoxic potential, including immunosuppressive, chemotherapeutic, and anti-infective drugs. TBI is also a risk factor for nephrologic complications.[19, 20] In our patients, 7.0% had permanently (>100 d after HSCT) elevated serum creatinine levels in follow-up exams. One patient (0.4%) briefly required dialysis for acute renal failure. In one prospective study by Kist-van Holthe et al. 2002, 11% of the patients developed chronic renal insufficiency after HSCT, a few percent more than in our patient-collective. Similar to this study, we also did not find irradiation to be a risk factor for chronic renal insufficiency.[21]

One of the gravest and ultimately lethal complications associated with HSCT is transplant failure. In our cohort, two transplants were rejected by the recipient. Both patients had undergone haploidentical HSCT and died despite re-transplantation, one 33 days post-HSCT of a gram-negative sepsis during complete aplasia, the other of transplant rejection with 90% blast cells on day +32 and rapidly progressive hyperleukocytosis on day +41. This patient died 47 days post-HSCT. These data is in accordance with the literature showing poor prognosis in patients with transplant failure.[22, 23]

In our cohort, 5.7% were diagnosed with a cardiomyopathy (all severity grade I). One of the main risk factors is the chemotherapeutic agent anthracycline.[24, 25] Due to the long 20- to 30-year latency period, long-term follow-up is required to screen for cardiomyopathies. Long-term survivors remain at a four times higher risk than the general population.[26] 11.4% of our patients had ECG abnormalities. Among the risk factors–as for cardiomyopathy–are anthracycline and irradiation of the thorax.[27–29] 80.8% of these patients received chemotherapy before transplantation. 10.5% of our patients had elevated blood pressure, significantly more of them allo-HSCT patients. 16.7% of these patients required drug therapy. GvHD and its treatment is a risk factor for hypertension. 76.2% of our allo-HSCT patients with an abnormal blood pressure had GvHD. In addition to GvHD, risk factors for abnormal blood pressure include infections, immunosuppressive agents, and especially CsA (Ciclosporin A), tacrolimus, and prednisone.[30–32] Our patients with abnormal blood pressure received CsA (45.8%), tacrolimus (12.5%), and prednisone (41.7%). At the conclusion of follow-up, 1.3% of our patients were receiving antihypertensives. Thrombosis/embolism affected 12.2% of our patients. Thrombosis was significantly more common in our allo-HSCT patients, the majority associated with catheters. A 2014 study showed CVC (Central Venous Catheter) to be a risk factor for the development of thrombosis: of the 20.0% of patients that developed thrombosis, 78.2% had a CVC.[33] Routine anticoagulation prophylaxis is not recommended for patients with CVC. Due to the risk of bleeding associated with thrombopenia, anticoagulation must be used on a case-by-case basis.[34]

Endocrine disorders are common late complications in HSCT patients: 50.7% of our patients had endocrine disorders, some of them among multiple concurrent disorders. The most common endocrine disorders involve the hypothalamic–pituitary-gonadal axis.[35, 36] Among our patients, 33.4% had abnormally elevated FSH (Follicle stimulating hormon / LH (Luteinizing hormone) levels. In a 2014 study, one third of HSCT patients had endocrine disorders[37] and 21.0% had growth disorders, including stunted growth (<3. percentile) and growth hormone deficiency. Other studies report growth disorder rates ranging from 20% to 80%.[38] The highest incidence in our cohort occurred in patients with CNS tumors and Lymphoma-patients, a rate attributable to the tumor site and high rate of irradiation in these patients.[39] Among our patients, 25.6% with growth disorders had undergone irradiation, 9.6% of them exhibiting a hyperthyroidism requiring substitution.

The trend toward longer survival times has increased the risk of second malignant neoplasms (SMN). An SMN was detected in 0.4% of our patients during follow-up exams. This patient with Hodgkin’s disease as underlying disease developed secondary myelodysplastic syndrome (MDS). In the literature, 1.4 to 6.9% of patients suffer from secondary neoplasia 10 years after HSCT.[40] The number of SMN rises (up to 15.0%) with increasing time after HSCT.[41] Because the follow-up period after HSCT in our cohort is limited for some of our patients, this number will certainly increase over time. Therefore, follow-up exams over a long time period are mandatory.

Abnormal audiometric results were observed in 11.4% of our patients. Risk factors for this are TBI, cranial irradiation, and medications with ototoxic potential like cisplatin, carboplatin, and high-dose aminoglycoside. No significant correlation between these risk factors and hearing loss were found in our analysis. Still, 23.1% of our patients with hearing loss received platinum-based drugs during conditioning. Punnett et al. reported a 44% rate of abnormal audiometric results–all of their patients had received platinum-based drugs.[42] Only 15.4% of our patients received platinum-based drugs, which possibly explains our low rate of hearing impairment.

Psychosocial disorders are common in children undergoing cancer therapy. Among these disorders are anxiety, stress, and depression. The literature reports rates between 5% and 40%.[43, 44] Of our patients, 12.2% needed child or adolescent counseling, 25.0% of them required medication. It can be assumed that the numbers would be significantly higher if unreported cases were counted. Follow-up exams should therefore focus not only on apparatus-based diagnostics but also on factors affecting patient quality of life and coping with the disease.

Among the most serious complications associated with allo-HSCT is GvHD, which is associated with elevated morbidity and mortality rates. Among our allo-HSCT patients, 59.5% developed an aGvHD, 22.2% a cGvHD. The literature reports rates of 10% to 60% for aGvHD and 20% to 50% for cGvHD in adult allo-HSCT patients.[45, 46]

The main risk factor for GvHD is an HLA disparity between donor and recipient cells. Among our patients, 21.3% developed an aGvHD after non-HLA-identical HSCT, 14.3% developed a cGvHD. 17.4% (n = 4) developed cGvHD following DLI or CD-34^+^-stem cell boost. However, entire mismatch (<9/10) transplanted patients received graft with a (partial) T-cell depletion. This might contribute to the relatively low GvHD rate. Other risk factors for GvHD are prior TBI, older age at time of HSCT, and male gender.[47, 48] In the present study, 32.0% of patients with aGvHD and 50.0% with cGvHD had undergone prior TBI. Prior graft manipulation reduced the risk of GvHD. However, in some circumstances–especially T-cell depletion and CD-34^+^-selection[49, 50]–this may elevate the recurrence risk and should be done on a case-by-case basis. The pathogenesis of GvHD is multivariate, often involving a combination of risk factors. This makes it difficult to determine a definitive cause.

Because pulmonary dysfunction often has a long latency period, long-term regular monitoring of patients at risk is needed. In the event of pathologic findings, bronchial infections must be prevented by inoculation against influenza and pneumococcal bacteria. The incidence of pulmonary late complications is much higher in adult HSCT patients than in pediatric patients due to adult smoking and a higher rate of GvHD. A preventive measure for children and adolescents after HSCT is absolute abstinence from nicotine.

In summary, in our retrospective analysis, we could calculate a complication rate of 74.2%. The present study was performed briefly after the last stem cell transplantation included in our analysis. In view of late effects, the median follow-up of 2.5 years seems relatively short. Given that, it seems reasonable and useful to resume the data collection at a later time because some complications (cardiomyopathy, pulmonary, or renal dysfunction) occur only after a longer period of time. However, by analyzing the subgroup of patients with a follow up period of five years and longer, we could not find significant differences compared with the entire cohort regarding the overall complication rate as well as the detailed analysis. Therefore, most of the relevant complications seem to occur during the first two years.

In specific clinical cases, a decision between autologous and allogeneic HSCT is key for a favorable prognosis. The primary disease, the stage of disease, and the condition of the organ allows a decision between the two types of HSCT.[51, 52] Further criteria for choosing allo-HSCT or auto-HSCT are patients’ overall health, comorbidities, performance status, and disease risk/status. In pediatric patients, the indication for stem cell transplantation mainly depends on primary disease and optimal donor and graft source. It is essential for a successful therapeutic process in stem cell treatment to perform it strictly according to therapy protocols [53]. As a rule, the earlier the diagnosis happens and the start of treatment is, the better the prognosis for late complications of HSCT is.

### Conclusion

In view of the expected longer survival time in children, HSCT-associated complications need to be recognized early on to lower morbidity and mortality rates. Therefore, beside the improvement of classic conditioning regimes, improved follow-up schedules, and a standardized transition to the medicine department seems to be necessary for enhanced transplantation success. The establishment of a tailored risk adjusted therapy with specific long-term follow-up schedule should be the focus on future treatment protocols. These might further improve HSCT success by preventing secondary morbidity to improve long-term quality of life and overall survival in pediatric HSCT patients. Nevertheless, HSCT is often the last resort of a therapeutic intervention with a curative goal. Therefore, (rapid) changes in disease-specific pre-transplant therapies will influence the outcome and should be kept in mind when interpreting data. Our retrospective data analysis points toward a crucial problem in the field of pediatric bone marrow transplantation, where prospectively and systematically collected, multicenter data about children receiving autologous or allogeneic HSCT is still scarce.

## Supporting information

S1 Table Raw data. (XLSX)

S2 Table Declaration of selected complications following allogeneic or autologous HSCT. (DOCX)

S1 Fig Statement of the ethic committee. (TIFF)
