# Supplementary material for: Early and late complications following hematopoietic stem cell transplantation in pediatric patients – A retrospective analysis over 11 years
Source: PLoS One. 2018 Oct 16;13(10):e0204914. doi: 10.1371/journal.pone.0204914 (PMC6191171; doi:10.1371/journal.pone.0204914)
Supplement: S2 Table — (DOCX) [file pone.0204914.s002.docx]

| Declaration of selected complications following HSCT: |  |
| --- | --- |
| TRM | All cases of mortality except progress or relapse of the underlying disease |
| CNS disorders | clinical symptoms of PRES or radiologic proved pathologies |
| Cardio-vascular complications |  |
| Cardiomyopathy | Diagnosis made by echocardiography (pathologic EF or dyskinesia) |
| ECG abnormalities | ECG abnormalities |
| Hyper - / Hypotension | Documented pathologic parameters requiring medication |
| Thrombosis / Embolism | Radiologic diagnosed pathology (ultrasound/CT) |
| Pulmonary complications |  |
| Acute respiratory insufficiency | Respiratory complaints and acute breathlessness: oxygen administration, high flow, CPAP or intubation was necessary |
| Renal insufficiency | Elevated creatinine levels >100 d after HSCT; acute renal failure requiring dialysis |
| Abnormal audiogram | High/low-frequency hearing loss, surdity, tinnitus, chronic tube dysfunction, |
| Endocrine disorders   - Growth - Thyroid disease - Gonads | Growth retardation (<3. percentile), reduced concentration of growth hormons (IG-BP3 / IGF)  Hypothyreoidism (latent / manifest) requiring medication  Hypo/hypergonadotropic Hypogondadism, ovarian-/testicular insufficiency (pathologic laboratory values |
| Psychosocial problems | In case conference of the Dept. of Child and Adolescent Psychiatry diagnosed diseases (anxiety / stress / adaption disorder, depression) requiring medication |
