# Supplementary figures and images for: Early and late complications following hematopoietic stem cell transplantation in pediatric patients – A retrospective analysis over 11 years
Source: PLoS One. 2018 Oct 16;13(10):e0204914. doi: 10.1371/journal.pone.0204914 (PMC6191171; doi:10.1371/journal.pone.0204914)

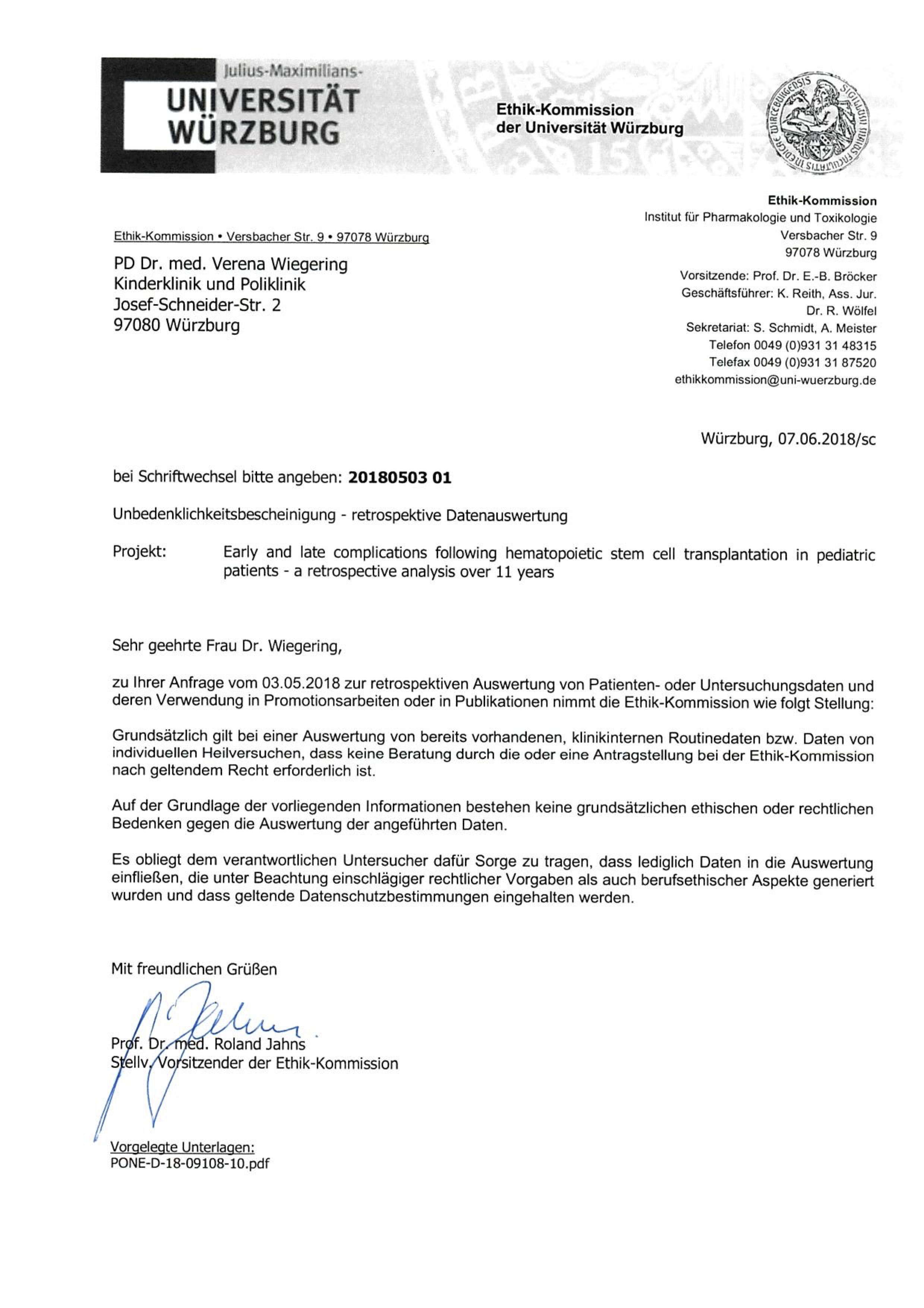

Supplement: S1 Fig — (TIFF) [file pone.0204914.s003.tiff]
